# Automating Risk Analysis of Software Design Models

**DOI:** 10.1155/2014/805856

**Published:** 2014-06-18

**Authors:** Maxime Frydman, Guifré Ruiz, Elisa Heymann, Eduardo César, Barton P. Miller

**Affiliations:** ^1^Computer Architecture and Operating Systems Department, Universitat Autónoma de Barcelona, Campus UAB, Edifici Q, Bellaterra, 08193 Barcelona, Spain; ^2^The Open Web Application Security Project (OWASP), 1200-C Agora Drive, No. 232, Bel Air, MD 21014, USA; ^3^Computer Sciences Department, University of Wisconsin, 1210 West Dayton Street, Madison, WI 53706-1685, USA

## Abstract

The growth of the internet and networked systems has exposed software to an increased amount of security threats. One of the responses from software developers to these threats is the introduction of security activities in the software development lifecycle. This paper describes an approach to reduce the need for costly human expertise to perform risk analysis in software, which is common in secure development methodologies, by automating threat modeling. Reducing the dependency on security experts aims at reducing the cost of secure development by allowing non-security-aware developers to apply secure development with little to no additional cost, making secure development more accessible. To automate threat modeling two data structures are introduced, identification trees and mitigation trees, to identify threats in software designs and advise mitigation techniques, while taking into account specification requirements and cost concerns. These are the components of our model for automated threat modeling, AutSEC. We validated AutSEC by implementing it in a tool based on data flow diagrams, from the Microsoft security development methodology, and applying it to VOMS, a grid middleware component, to evaluate our model's performance.

## 1. Introduction

Software supports the information structure of businesses and governments worldwide. The growth of the Internet and networked systems has implied an increase of threats and challenges for software development companies. To address this issue security activities are increasingly being introduced into the software development lifecycle to reduce the number of software defects earlier in the software cycle. Reducing software defects earlier in the software lifecycle offers two main advantages; first it lowers the cost of fixing the software and second it limits the risk of deploying insecure software to users.

There are currently three high-profile approaches to the development of secure software (detailed in Section 2), the* OWASP comprehensive lightweight application security process (CLASP)* [1],* McGraw Touchpoints,* [2] and the Microsoft security development lifecycle (SDL) [3].

All of these secure development methodologies share one essential risk analysis activity, called* threat modeling *[4], used to guide the following steps of the process. In this activity, the architecture of the system is represented and analyzed, generally prior to the implementation, to identify potential security threats to the system and to select appropriate mitigation techniques to address them.

Unfortunately, this activity either must be performed by* security-aware developers* or requires a* core security team* as most developers are not used to thinking and acting as professional attackers [5], nor do they have the necessary security expertise to imagine sophisticated attack scenarios [6] and mitigation strategies. This need for security expertise adds a significant cost to secure software development which reduces the chance that it will be used in many software projects.

In this paper we address the problem of the security expertise required for risk analysis. We created a model, AutSEC (automated security expert consultant), that automates the risk analysis process. The purpose of AutSEC is to enforce* security by design*, where threats are mitigated early in the development process, and automate all security operations of the threat modeling process to allow non-security-aware engineers to develop secure software.

To validate AutSEC we implemented the model in a tool that integrates with the Microsoft SDL methodology. This implementation is compatible with the Microsoft threat modeling process and tool, facilitating its integration in development environments where SDL is already deployed.

This paper makes the following contributions.

- Two new data structures,* identification trees*, which contain information to identify threats in software design models, and* mitigation trees*, which classify threat countermeasures by their costs.
- A model, AutSEC, relying on these two data structures, that purges the less relevant threats according to the business policies and estimates the mitigation techniques of least effort that adhere to the software specification.

The rest of this paper is organized as follows. Section 2 describes current methodologies used for threat modeling.  Section 3 describes the input expected by our tool in addition to the SDL standard.  Section 4 describes the methodology of the model used to automate threat identification, sort risks, and compute least effort countermeasures.  Section 5 presents experimental results obtained by applying our tool to the grid middleware component VOMS Admin.  Section 6 analyses the experimental results. Finally Section 7 concludes our work.

## 2. Related Work

There are currently three widely deployed methodologies for secure application development. Each of these methodologies has the same purpose, that is, to detect and eliminate security threats to applications throughout the development lifecycle of the application. This activity begins during the architectural design of the application and ends after the application has been tested and deployed.

The* OWASP comprehensive lightweight application security process (CLASP)* is a set of processes that can be integrated into any software development process and is designed to be both easy to adopt and effective. This makes CLASP more suitable for small organizations. It takes a prescriptive approach, documenting activities that organizations should be doing, and provides an extensive wealth of security resources that make implementing those activities reasonable.

The* McGraw Touchpoints*, a methodology that involves explicitly pondering the security situation throughout the software lifecycle. This means identifying and understanding common risks, designing for security, and subjecting all software artifacts to thorough, objective risk analysis and testing. It is based on industrial experience gathered over many years.

The* Microsoft security development lifecycle (SDL)* is a software development security assurance process consisting of practices grouped in seven phases: training, requirements, design, implementation, verification, release, and response.

All three methodologies share a common activity called* threat modeling* where the software under development is modeled. This model is then used by security experts to identify potential threats to the software and how to best mitigate them. This is a crucial step in secure application development as it orients the security efforts that will be deployed throughout the applications development lifecycle.

Our proposal reduces the reliance on security experts by* automating the threat identification and mitigation step*. Our model was developed to be generic; however our implementation used to validate AutSEC is compatible with the Microsoft SDL methodology. This choice was made as the SDL methodology offers a modeling tool that meets the requirements of threat matching, while having the flexibility to add custom annotations used by our model to refine the analysis (further described in Section 4).

Our model relies on a knowledge base called* attack patterns* to perform threat identification. This knowledge base is composed of threats that AutSEC is capable of identifying. Each threat in our knowledge base is represented by an* identification tree*, a* mitigation tree,* and ranking information. The* identification tree* is used to identify potential threats based on the software model and is based on the work found in [7]. The* mitigation tree* represents all the possible countermeasures that can be used to address a threat. Mitigation trees are a new concept to list and rank possible countermeasure but its representation is based on concepts introduced by attack trees.

## 3. Software Design Modeling

There are several approaches used to represent software designs for security purposes [8]. As explained in Section 2, our implementation of the AutSEC model is aimed at automating the widely used threat modeling [9] process of the Microsoft security development lifecycle (SDL), which uses data flow diagrams (DFDs) to represent the software architecture. To perform the modeling, Microsoft provides analysts with a modeling tool [10]; our implementation is based on the output of this tool and only requires a few specific additions to the original diagrams.

Our implementation expects the system to be represented as defined in the threat modeling process, which consists of data flows, data stores, processes, and trust boundaries to build the DFDs [3]. In addition, it is expected of the developers to make three small additions to elements in the form of attributes.

- *Asset value* represents the value as a resource, {high, medium, and low}, of a DFD element; for example, a server might be valued as high, while the log files might be valued as low. This is based on the potential damage that would result in the resource being compromised.
- *Languages* are programming language used, for example, Java or C++.
- *Frameworks* are frameworks or other external software libraries used, if any, for example, CSRF Guard or ESPAI.

The* asset value* attribute must be defined for each DFD element, but* Languages* and* Frameworks* must be defined only for processes. This information will be used to refine the results in the threat identification and risk sorting steps. Since our tool is implemented on top of the current threat modeling process, it is important to maintain compatibility with the current SDL tool. The addition of the new attributes is performed by utilizing the assumption feature of SDL and allows native integration.

**Figure 1:**
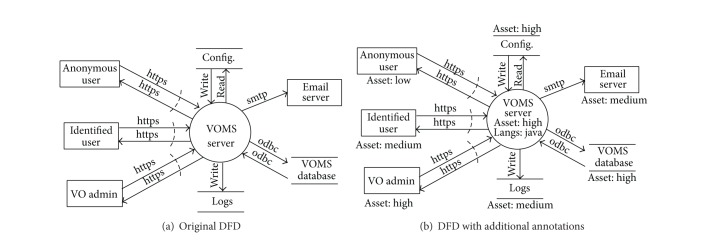
Original DFD compared to annotated DFD.

An example of the required additions is shown in Figure 1.  Figure 1(a) shows the original DFD modeled according to SDL.  Figure 1(b) shows the same model as well as the new added attributes required by AutSEC. In this example, each DFD element of Figure 1(b) has now been assigned an asset value and the only process, VOMS server, has been assigned a language.

The* asset value* is determined based on the damage that can be done if the resource is compromised. The VOMS server was classified as a high value asset, the mail server was classified as a medium value asset, and the log files are classified as low value assets. In the case of VOMS, compromising the main server would allow an attacker to compromise the operations of VOMS while obtaining the log files would at best disclose certain private information.

## 4. Methodology

The aim of our model, AutSEC, is to automate the threat modeling activity so that non-security-aware developers can perform secure development. The model described in this section takes the diagrams produced by the developers during the requirements and design phase of their software and produces documentation that will identify threats and describe how to mitigate the threats throughout the software's lifecycle.

AutSEC is a 4-step process whose result is to generate three detailed reports, one for each relevant software development activity; these are the design, implementation, and verification reports.

The design report discusses architectural and design decisions that can mitigate or eliminate potential threats. The implementation report shows how to implement certain features in a secure manner. The verification report combines all the threats contained in the design and verification reports and details how to assert that each threat has been properly mitigated. These three reports reflect the stages of the development lifecycle.

Since our model is aimed at developers regardless of their security expertise, we have taken great care as to limit interaction with the developers. When our process requires inputs, the inputs take the form of specific questions that a developer is able to answer, that is, business requirements, implementation details, and general mechanics, about the software he is developing and in the form of a multiple choice or polar question (yes or no). For the same reason the documentation produced as output of our model is presented with all the necessary information to understand each threat and its mitigation technique.

**Figure 2:**
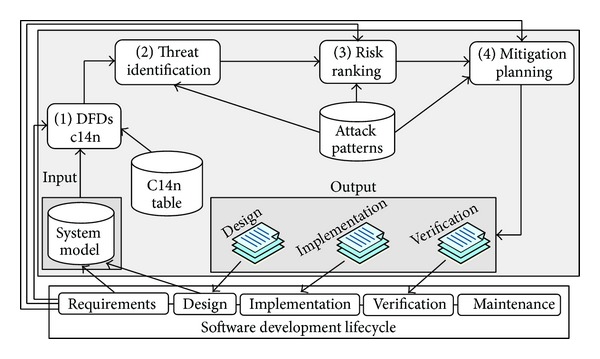
General architecture diagram of our approach.

The input to our model is the DFD produced by the threat modeling tool according to Section 3, and the outputs are the three detailed reports mentioned above. AutSEC is a 4-step process as shown in Figure 2.

1. *Data flow diagram canonicalization* to interpret the labels of user-defined elements of the diagram.
2. *Threat identification* to identify threats relevant to the diagram.
3. *Risk ranking and threat purging* to prioritize threats according to risk and dismiss threats depending on business requirements.
4. *Mitigation planning* to propose countermeasures for the discovered threats to the developers that are compatible with their requirements.

The combination of these four steps results in the threat evaluation of the user application. To perform the threat evaluation we use two knowledge bases.

 
*C14n Table:* C14n is the canonicalization table that contains the information used to map unknown user labels of the diagram to known values.
 
*Attack Patterns:* the attack patterns are a collection of information over each threat that contain the* identification tree* used to identify the threat, the* risk attributes* used to rank the threat, and the* mitigation tree* used to mitigate the threat.

The information concerning threats used to build the* attack patterns* was gathered from several relevant security sources and standards, such as* Common Attack Pattern Enumeration and Classification (CAPEC)* [11],* Common Weakness Enumeration (CWE)* [12], and* Open Web Security Project (OWASP)* [13] amongst others. These databases contain generic information that can be applied to any software as long as it is modelled with the methodology described in Section 3.

The following subsections describe each of AutSEC's 4 steps in detail.

### 4.1. Data Flow Diagram Canonicalization

**Figure 3:**
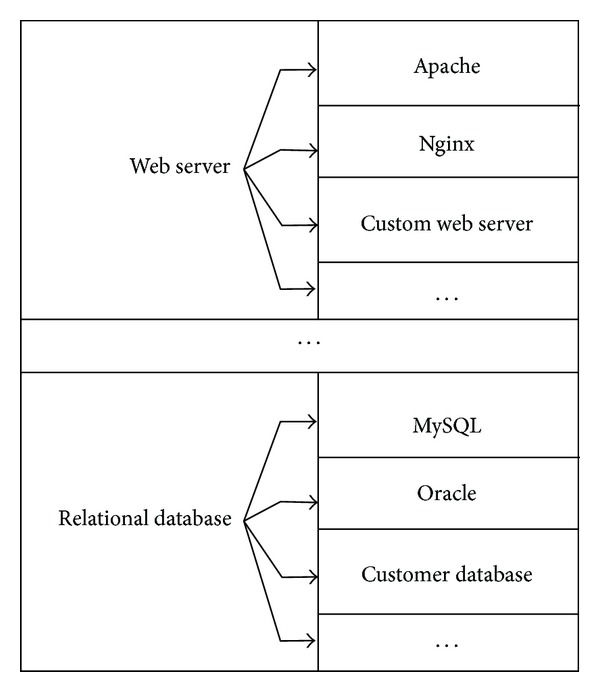
Example multimap mapping.

The first step of AutSEC is the data flow diagram canonicalization; this serves to map unknown user-defined labels to ones that can be automatically interpreted, for example, the identification of a user defined entity called* Apache* as a* web server*. This is accomplished using a data structure called* MultiMap* that allows the mapping of a set of values to a single key to build a* canonicalization table*; see Figure 3.

The purpose of canonicalization is to obtain specific information about the elements contained in the diagram of the application. This increases the precision of the threat identification and reduces the amount of generic threats reported.

While this process performs relatively well, it is not possible to anticipate every declination that can be given to DFD elements. This is addressed by the questioning phase of AutSEC, where unmapped elements can be refined by the developers. This gives flexibility to the tool both in terms of modeling restrictions as well as usability during the modeling process, only asking for refinements when interpretation has failed and learning from those refinements for further projects.

To interpret string attributes, each named element defined by the developer in the data flow diagram (DFD) is compared with the values contained in the MultiMap. If the mapping is successful then the label of the DFD element is replaced by the mapped key. Otherwise, a number of possible keys are presented to the developer for the unknown DFD element. This comes in the form of a list of generic items that are common in software development, for example, relational databases, web servers, and user interfaces. If one of these items is selected by the developer, the new value is added to the* canonicalization table* and its mapping key is assigned to the element of the DFD. If there were no suitable mappings, a generic value is assigned to the element and it will be treated as a generic element.

The resulting canonicalized DFD serves as input to the second step of the process.

### 4.2. Threat Identification

The second step of AutSEC is to perform threat identification based on the information contained in the canonicalized DFD.

This step is the core of the analysis; all further steps rely on the accuracy of the threat detection. To identify threats, a set of trees,* identification trees*, were designed. Each threat defined in the* attack patterns* contains an* identification tree* which is used to determine if the threat is relevant to the DFD.

**Figure 4:**
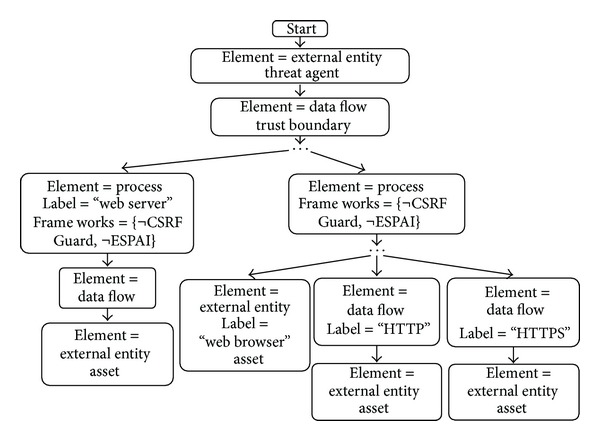
Identification graph of CSRF threats.

Each branch of an* identification tree* represents a subgraph to be matched in the DFD. If the tree is matched with the DFD, it means that the threat is relevant. Each node in the tree represents an element of the DFD and can indicate additional attributes required for the match to be valid. These attributes can either be a requirement or indicate that a specific element cannot be present in the DFD for the match to be successful.  Figure 4 shows an example of* identification tree* for cross-site request forgery (CSRF) threats.

As shown in the figure, CSRF is a possible threat when there is data crossing a trust boundary (attack surface (the attack surface is the collection of interaction points with a software available to an attacker) [14]) to an HTTP server and the process that handles the HTTP request does not use specific frameworks against CSRF threats. If an anti-CSRF framework was used, it would indicate that the threat is mitigated making the threat irrelevant. This is represented by a key value pair, and the value has a “¬” symbol to indicate the negation in the match. In addition, each threat defines the* Threat Agent*, the component carrying out the attack, and the* asset*, the component compromised. In this case the threat agent is an external entity and the asset is a resource of value present on the web server. Certain threats can also require specific canonical labels; here the requirement for a successful CSRF attack is the presence of a web server.

The tree of Figure 4 is relatively generic for client-side threats of web technologies and can easily be reused for other types of threats.

Each threat identified in this step is added to a list of threats potentially affecting the software. This list serves as input to the next step of the process.

### 4.3. Risk Ranking and Threat Purging

The third step of AutSEC is to rank the list of identified threats by risk; this serves two purposes.

First purpose is the sorting of discovered threats to be able to prioritize the order in which they should be addressed. Second purpose is to allow the purging of threats; while certain potential threats can be present in a system, they might be considered too unlikely to occur or to have a very insignificant impact. For this purpose the user can set a threshold to eliminate certain threats based on business security policies.

The sorting is performed using the US National Security Telecommunications and Information Systems Security Committee [15] definition of risk in

(1) $$\begin{array}{c} \text{Risk}=\text{Likelihood}\times \text{Impact}. \end{array}$$

The* likelihood* of exploitation value is taken from the CAPEC security source; its potential values are* very high (*1),* high (0.75)*,* medium (0.5)*, and* low (0.25)*. The* impact* is calculated as shown in

(2) $$\begin{array}{c} \text{Impact}=\text{Asset}\times \text{ThreatAgent}\times \text{CIA}\;\text{Impact}. \end{array}$$

The* asset* is defined by the developers, as explained in Section 3, and the* ThreatAgent* is the inverse of the* asset*. If a component is a high value asset, it will be a low value threat agent. For instance, if the* asset* is very high such as a database containing confidential information or an administrator of the system, it implies that it is a highly trusted component and the risk of suffering an attack from it is low. The possible values for the* asset* and* ThreatAgent* are* high (1.2)*,* medium (1.0)*, and* low (0.8)*. Combining this information the* CIA impact* is computed as shown in

(3) $$\begin{array}{c} \text{CIA}\;\text{Impact}=\text{Conf}\;\text{Imp}+\text{Int}\;\text{Imp}+\text{Avai}\;\text{Imp.} \end{array}$$

The* confidentiality*,* integrity,* and* availability* impact information are gathered from the attack patterns of CAPEC. Their value can be* high (0.33)*,* medium (0.22)* or* low (0.11)*. This puts risks in a range of approximately 0.05 and 1.45.

Using this ranking each threat identified in the previous step is ranked according to risk. The choice is then offered to the developers to elect a threshold; a threshold is a value from 0 to 1.5. Threats ranked below this threshold will be purged and will not be considered for the mitigation planning.

The purged list of threats contains only those that scored above the threshold; this is the input to the final step of the process.

### 4.4. Mitigation Planning

The final step of AutSEC is the production of the results that will be used by the software developers for secure development. The results take the form of reports that address each threat that was detected.

These reports are separated into three categories. The first addresses the design activity of the development and indicates architecture consideration to mitigate threats. The second addresses the implementation activity where specific guidelines are given to mitigate the threat. The third report concerns testing and serves as a fail-safe measure to ensure that all the detected threats were properly mitigated.

The architecture and implementation reports contain countermeasures for each identified threat. There are usually a number of possible countermeasures available to address a threat. Each of these countermeasures has implications, whether in terms of effort or as limitations to the software. For example, while removing all input to a software might be an efficient and effortless way to mitigate most threats, it is unlikely to conform with business requirements.

To address the election of the proper countermeasure, a new data structure was defined, called* mitigation tree*. The purpose of* mitigation trees* is to determine the mitigation of least effort, that is, those that require the least monetary and/or time investment, to address the threat while conforming to the business requirements. If the countermeasure is not suitable, alternatives will be proposed that are more flexible but incur a higher effort.

Attack trees have been widely used by the community to represent attacks in a similar way as attack patterns do. Its root is the goal of an attacker, and each branch contains the set of actions that an attacker must carry out to achieve the goal at the root.* Mitigation trees* are similar however with a constructive rather than destructive intent. The root of the* mitigation tree* is the goal of mitigating a determined threat; each branch contains the set of software specifications or features, for the design and implementation activities, needed to accomplish the goal of the root. In addition, each feature contains an* estimated cost* associated to its implementation. This information is stored for each mitigation in the attack pattern of the threat.

**Figure 5:**
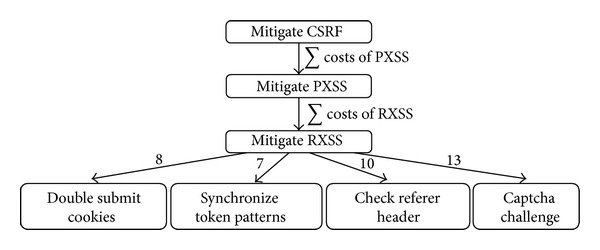
Mitigation tree of the CSRF attack pattern.

Figure 5 shows the mitigation tree of CSRF attacks. It shows that to mitigate CSRF threats it is necessary to first mitigate all persistent cross-site scripting (PXSS) and reflected cross-site scripting (RXSS) threats and then offers four subbranches that represent different mitigation techniques. Each node or software specification of the attack tree has an estimated cost. This cost is calculated by using an* expert judgment* approach [16], where the security expert that builds the tree establishes a relative cost for each software specification using his past experiences as criteria.

During the mitigation planning, a set of polar questions are presented to the developers concerning design specifications, which are not shown in the DFD, and are relevant from a security point of view. These questions aim to identify whether certain threats are already mitigated and need not appear in the mitigation planning.

Since the purpose of this method is to emphasize* security by default* and* security by design*, it might be possible that certain countermeasures degrade the usability of the system, or that they are incompatible with the business requirements. When a certain countermeasure implies limitations, these are presented to the developers to ensure that they are acceptable. When a countermeasure is rejected, the* least effort* mitigation is recomputed excluding the incompatible mitigation. This process is repeated until a suitable countermeasure has been found for each threat.

While the verification and design reports indicate the measures to be taken to mitigate threats, this does not guarantee their proper implementation. This is addressed by the verification report, where each threat is set to be tested. If the threat is not successfully avoided during the elaboration of the architecture and the implementation, it will be detected when carrying out the penetration testing actions of this activity. The verification report contains for each threat the testing activities, some example exploit code, and relevant references.

Due to unclear boundaries between design and implementation [12], we define the boundary here as follows. If it can be modeled in UML, it corresponds to the design activity, otherwise to the implementation.

## 5. Experimental Results

To evaluate the validity of AutSEC's approach, the distributed grid middleware component VOMS was used as case study using our tool that implements AutSEC. VOMS is a grid middleware that manages virtual organizations and user certificate attributes that will later be used by other middleware systems to take authorization decisions.

This section shows how the DFD diagram of VOMS Admin, a component of VOMS, is produced, how this diagram is processed, and the reports that result from this diagram. The examples provided in this section limit themselves to one element of VOMS and a specific threat, the 3 full reports containing all the threats and the DFD compatible with our tool are available in [17].

### 5.1. VOMS Admin DFD Diagram

**Figure 6:**
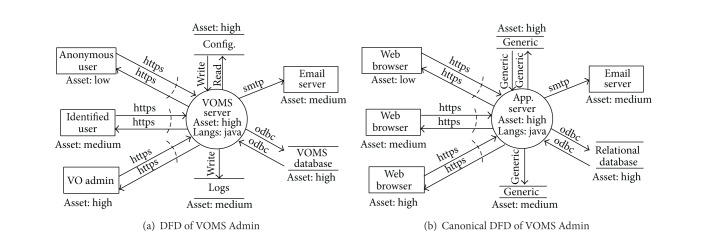
VOMS Admin parsing.

Figure 6(a) shows the data flow diagram of VOMS Admin that was built as described in Section 3 using the threat modeling tool, which is the main manual phase of the assessment. After the diagram has been built, limited interaction is required to choose the desired level of security and the willing balance between security by default and usability.

### 5.2. VOMS Admin DFD Canonicalization

Figure 6(b) shows the canonicalized diagram produced from the original diagram. A few labels could not be automatically mapped, like the VOMS server, configuration files, and logs, as they do not appear in the canonicalization mapping table. These were set by the developers during the polar question phase where the VOMS server was specified to be an app server and the other resources were assigned as generic entities.

It is from this canonicalized DFD that the threat identification will be performed.

### 5.3. VOMS Admin Threat Identification

During the threat analysis step, the subgraphs of attack patterns are matched with the canonical DFD of Figure 6(b) to find the potential threats to the system.  Table 1 shows each detected vulnerability according to the DFD and the report in which it appears.

As can be seen, a wide range of potential vulnerabilities are detected that correspond to the DFD. The threats are separated between the design and implementation report, depending on where it is most appropriate to mitigate the issue. The verification report covers all detected threats to ensure that they have been properly addressed.

The cross-site request forgery (CSRF) threat is used in this document to provide a complete example of AutSEC's process.

**Figure 7:**
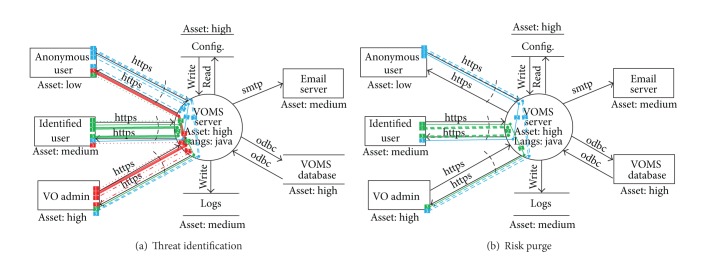
VOMS Admin risk analysis.

The detection of the CSRF threat results from the matching of the subgraphs shown in Listing 1 and represented in Figure 7(a).

As can be seen, a number of different types of potential CSRF attacks are identified for each type of web user, that is, anonymous, identified, and admin, can perform a CSRF attack that can target every type of user. These represent all the potential CSRF threats to the software.

Once all the potential threats are identified, the next step is to purge those that are least likely to have a significant impact.

### 5.4. VOMS Admin Risk Ranking

The risk ranking step of this process is where the threats detected in Listing 1 are sorted according to their potential threat.  Listing 2 shows the results of the ranking using the CSRF example and is represented in Figure 7(b).

As can be seen, the most likely and damaging scenario for a CSRF attack is the one where an unprivileged user attacks an administrator while the least likely scenario is the one where an administrator attacks an anonymous user.

In Listing 3, the developers are asked for the threshold that is relevant to the activity. With a threshold of 0.5 only the most relevant CSRF attacks are kept discarding threats that score below the threshold.

### 5.5. VOMS Admin Mitigation Planning

Finally, before the final reports can be produced, the existing mitigation measures have to be identified and the countermeasures proposed by AutSEC have to be evaluated by the developers for compatibility with the requirements.

As described in Section 4.4, this is done using a set of polar questions regarding the design of the system to refine the results. This is shown in lines 1 to 3 of Listing 4. After these questions have been answered, the countermeasures of* least effort* are estimated and software developers are asked if they are compliant with their software specification. If not, they are recomputed until a balance between security and usability is reached. This is shown in lines 5 to 15 of Listing 4.

This is the final step of the process and the reports are then generated containing the mitigations that have been approved.

It is interesting to notice that this approach has detected that to mitigate a CSRF threat it is first required to mitigate all PXSS and RXSS threats. For this reason, it asked in line 9 of Listing 4 if it was possible to only allow alphanumeric characters in the HTML forms. Engineers answered “n” (no) because special characters are required in some fields. Therefore, mitigations were recomputed resulting in not only a more permissive but also a more expensive solution which is to HTML encode user supplied data before displaying it back to the web interface.

The mitigation choices are included in the final reports; these reports detail every threat detected that scored above the ranking threshold, the chosen mitigation technique that corresponds to the business requirements, and links that further describe the threat and their possible countermeasures.

## 6. Experimental Results Validation and Discussion

### 6.1. Validation

In order to validate our approach we compared the threats reported by our tool with the list of known VOMS vulnerabilities. The information on vulnerabilities affecting VOMS was gathered during the security audit carried out by our team, using the manual* First Principles Vulnerability Assessment* [18] methodology, as well as the collection of all the vulnerabilities reported by the community over the 2011–2014 period shared by the VOMS development team. All of the vulnerabilities discussed in this paper have been fixed and disclosed.

A summary of these vulnerabilities can be found in Table 2.

AutSEC was applied to VOMS a posteriori; that is, the process was applied to VOMS after its release and 10 years of activity. Although the main purpose of AutSEC is to be applied during the elaboration and development phases of software, it would be difficult to quantify the validity of our approach without perspective on the security issues that arise after software has been released for a length of time.

There are 3 crucial elements that affect the validity of this tool; they are the quantity of information contained in the* attack patterns*, the quality of the matching, and the accuracy of the ranking. The quantity of information contained in the* attack patterns* increases the security knowledge of the tool. The quality of the matching defines whether the knowledge contained in the attack patterns can be correctly put to use to identify threats. And the accuracy of the ranking is what allows prioritizing the focus of the security effort and discarding irrelevant threats. A tool which can only detect a single vulnerability but with 100% accuracy is of limited use, and so is one that detects every threat incorrectly. A useful security tool must strike a balance between those three factors.

Similarly to other automated security tools, it is of critical importance to present concise information to the developers that cover the widest array of significant risks, while limiting the amount of irrelevant information. This is traditionally called the ratio of false positives, threats that are reported but do not impact the system, to false negatives, threats that do affect the system but are not reported. The value of an automated tool to the developers is linked to the ratio of false positives to false negatives [19] as not reporting a potential vulnerability leads to a sense of false security, but reporting too many irrelevant vulnerabilities can be just as harmful as it conceals true threats.

In accordance with the definition of validity expressed above, this experiment was conducted using a vulnerability database that was not specific to VOMS but contained a variety of vulnerabilities from different programming languages and technologies. The following sections analyse the results provided by our tool in terms of successfully identified threats, threats that were not identified (possible false negatives) and threats that were identified but have not been reported as affecting VOMS (possible false positives).

### 6.2. Successfully Detected Threats

From the identified potential vulnerabilities of VOMS, 4 real vulnerabilities match our tool`s predictions. These are the 2 persistent cross-site scripting (PXSS), 1 cross-site scripting (XSS), and 1 cross-site request forgery (CSRF) vulnerabilities.

Analysis of these vulnerabilities shows that the mitigation techniques proposed by AutSEC would have successfully neutralized the threat and thus prevented the vulnerability. The verification fail-safe mechanism of AutSEC, in case the implementation is not correctly carried out, was also analyzed and it offered sufficient information to identify the vulnerabilities found in VOMS.

This result shows that our tool was successful in identifying the threat and offers useful mitigation techniques, and later in the application lifecycle it offers useful and relevant information to guarantee that the threat had been addressed. Early detection of vulnerabilities is the original purpose of AutSEC as it limits the financial impact of fixing vulnerabilities after deployment, as well as the impact on the software's users.

### 6.3. Undetected Threats

Considering the vulnerabilities found in VOMS and not reported by our tool, 2 vulnerability categories appear.

First category is vulnerabilities that lie outside of the scope of automated assessment. This category has been summarized as business-specific vulnerabilities, where the vulnerability is the result of improper implementation of domain specific requirements. While these are considered vulnerabilities, they are in no way related to the architecture or technology of the application and therefore cannot be detected using AutSEC's methodology. An example of this for VOMS is the incorrect check of certain certificate attributes; while this possesses a security threat to VOMS users, it is entirely related to the domain requirements.

The second category is vulnerabilities that lie within the scope of automated assessment. In the case of VOMS there are 2 vulnerabilities that enter this category,* DoS* attacks and* insecure third party library linking*. These vulnerabilities could be added to AutSEC's* attack patterns*. In our current implementation these types of vulnerabilities have not been included in the* attack patterns* as they are not due to specific architectures.* DoS* vulnerabilities, for example, are a generic issue that can affect any host providing service on a network.

This poses a complex issue between exhaustiveness and relevance, as reporting too many generic vulnerabilities can hurt the visibility of those specific to the system, as well as the difficulty of properly ranking these vulnerabilities without further information not found in the current architectural diagrams. These types of vulnerabilities are easily identified in our current representation, that is, a threat whose attack tree requires the presence of only a single DFD element to be detected.

This is a subject that will be explored in the continuation of this research; one option currently under review is to add a fourth type of report that covers generic vulnerabilities for each technology used in a project.

### 6.4. Detected Threats Not Found in VOMS

There were a number of vulnerabilities reported by our tool for which no matching vulnerability was found in VOMS, as can be seen by comparing Tables 1 and 2. For example, SQL injections were identified as a potential threat but have not appeared in VOMS.

After analysis, the reported potential threats are considered to be relevant to the architecture of VOMS, they are not false positives, and their mitigation would benefit the VOMS software. That is to say, our team acting as security experts auditing VOMS would have explored whether these vulnerabilities were present or not as they are likely to occur and are potentially damaging.

Considering that the threats are relevant to the architecture, 3 possibilities exist to explain why they did not appear in VOMS. First, the proper mitigation techniques were implemented by the VOMS team. Second, implementation details of VOMS make this threat a nonissue even if the threat was present. Third, vulnerability exists but has not been uncovered.

Regardless of the reason why these vulnerabilities were not uncovered within VOMS, these threats are considered to be relevant to the architecture of the VOMS software, and therefore their reporting is considered valid.

## 7. Conclusions

In this paper, we addressed the problem of security expertise needed to perform risk assessments by automating the threat modeling process. By allowing nonsecurity developers to perform threat modeling we aimed at reducing the cost of secure development, making it more available.

To this end, we modeled a new data structure called* identification tree* that can be used to identify threats in software designs. We also designed a new model to describe countermeasures of threats called* mitigation tree*, which classifies the set of software specifications that are required to mitigate a specific threat. These data structures, along with ranking information over threats, were combined in a knowledge base called* attack patterns*.

In addition, we designed a new model, AutSEC, to automate threat modeling relying on the information contained in the* attack patterns*. We implemented this model in the form of an automated tool that works on top of the current Microsoft threat modeling methodology. AutSEC uses the* identification trees* to find the potential threats of a given software model. It purges irrelevant threats according to the developers business policies. And finally, it uses the* mitigation trees* to compute the software specifications of least effort needed to mitigate the detected threats during the development lifecycle.

The resulting least effort mitigations are directly related not only to* security by design* but also to* security by default*. This allows AutSEC to reach the willing balance between usability and security by default by asking the developers if the computed features are in good standing with their requirements. If not, they are recomputed rejecting those that do not comply.

The output of applying the AutSEC model comes in the form of 3 reports: one for the design activity, which contains the architectural modifications needed to be carried out in the system, another one for the implementation phase, which contains implementation details to avoid the threats, and a final report for the verification phase containing a set of actions that are needed to be carried out to verify that detected threats were properly mitigated. These reports were designed so that any security-unaware developer can carry out their recommendations, which are written in terms that developers are accustomed to and provide ample resources in the case further information is required.

Our implementation of AutSEC was designed to be compatible with the current threat modeling tool distributed with the Microsoft SDL methodology. This offers the advantage that it can be easily integrated into development teams who already make use of the Microsoft methodology with minimum modifications to their software models in order to make them compatible with AutSEC's extended SDL attributes.

The experimental results of our tool were validated using the grid middleware component VOMS Admin. The results show that our tool is capable of detecting threats and offers the appropriate mitigation techniques. We have shown how the use of AutSEC during the development of VOMS Admin would have allowed the early detection of certain vulnerabilities. This has the advantage of limiting the financial impact of vulnerabilities, without requiring software developers to be trained in security and eliminate the impact on the software's users.

Further research on automated tools will focus on expanding AutSEC's vulnerability coverage while maintaining a high level of accuracy in their detection and will look into new ways of presenting additional threat information to the developers without undermining the quality of current reports.

## Figures and Tables

**Listing 1:**
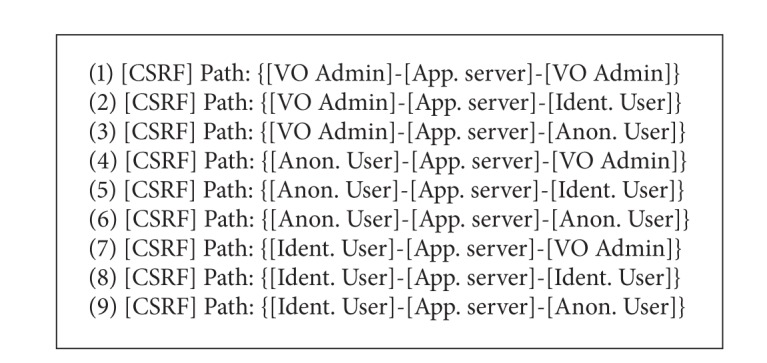
VOMS Admin CSRF threat identification.

**Listing 2:**
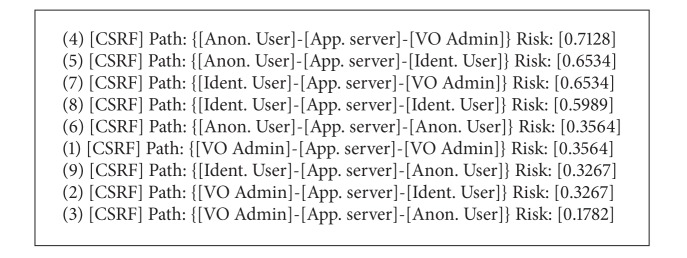
CSRF risk sorting threats.

**Listing 3:**
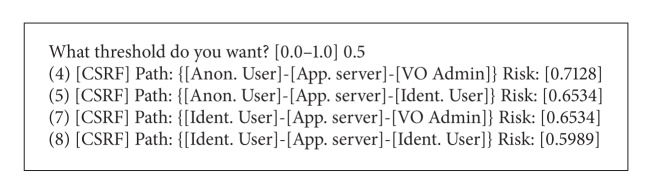
Sorted CSRF risk purging threats.

**Listing 4:**
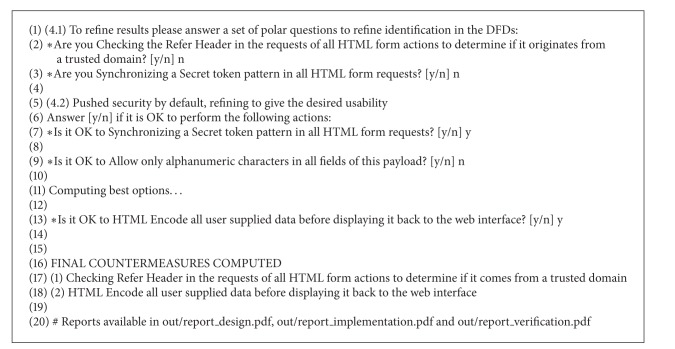
VOMS Admin mitigation planning.

**Table 1:** Vulnerabilities reported to be found in corresponding AutSEC reports.

Design report	Implementation report	Verification report
Cross-site request forgery (CSRF)	Time of check to time of use	Cross-site request forgery (CSRF)
Insecure cryptographic storage	SQL injection attacks	Insecure cryptographic storage
	Reflected cross-site scripting (RXSS)	Time of check to time of use
	E-mail headers injection	SQL injection attacks
		Reflected cross-site scripting (RXSS)
		E-mail headers injection

**Table 2:** VOMS vulnerability summary.

Vulnerability type	Count
Persistent cross-site scripting (PXSS)	2
Business specific	2
Cross-site scripting (XSS)	1
Cross-site request forgery (CSRF)	1
Denial of service (DoS)	1
Insecure third party library linking	1
